# A Phase II Clinical Trial of Chemotherapy Rechallenge with or without Targeted Therapy in Refractory Metastatic Colorectal Cancer

**DOI:** 10.32604/or.2026.084378

**Published:** 2026-08-13

**Authors:** Chenchen Wang, Mingzhu Huang, Wenhua Li, Xuedan Sheng, Xiaoying Zhao, Xiaodong Zhu, Zhiyu Chen, Zhe Zhang, Haiming Li, Weijian Guo

**Affiliations:** 1 Department of Medical Oncology, Fudan University Shanghai Cancer Center, Shanghai, China; 2 Department of Oncology, Shanghai Medical College, Fudan University, Shanghai, China; 3 Department of Radiology, Fudan University Shanghai Cancer Center, Shanghai, China

**Keywords:** Colorectal cancer, refractory disease, rechallenge therapy, clinical trial

## Abstract

**Background:** Patients with refractory metastatic colorectal cancer (mCRC) face limited treatment options after failure of standard therapies. This single-arm, phase II study aimed to evaluate the efficacy and safety of rechallenge strategies using previously effective regimens in late-line mCRC. **Methods**: Patients who progressed after ≥2 lines of prior chemotherapy, with a prior progression-free survival (PFS) ≥4 months and a ≥4-month treatment-free interval on that regimen were enrolled. Patients received rechallenge chemotherapy (oxaliplatin-, irinotecan-, or raltitrexed-based) with or without targeted agents (bevacizumab or cetuximab). Primary endpoint was investigator-assessed PFS. Secondary endpoints included objective response rate (ORR), disease control rate (DCR), overall survival (OS), and safety. **Results**: Forty-three patients were enrolled (31 received chemotherapy plus targeted agents; 12 received chemotherapy alone). One patient discontinued treatment, leaving 42 patients evaluable for tumor response. The median PFS and OS were 3.97 months (95% CI: 2.46–5.48) and 13.03 months (95% CI: 9.68–16.38), respectively, while the ORR and DCR were 2.4% and 61.9%. Subgroup analysis showed that patients receiving chemotherapy plus targeted agents had higher DCRs (80.0% for bevacizumab-based regimens and 72.7% for cetuximab-based regimens vs. 18.2% for chemotherapy alone;) and longer median PFS (4.17 and 4.50 months vs. 1.57 months, respectively). Grade 3 or 4 adverse events were reported in 39.5% of patients, with no severe adverse events or treatment-related deaths observed. **Conclusion**: Chemotherapy rechallenge strategies, particularly when combined with targeted agents, demonstrated promising clinical activity and acceptable safety in selected heavily pretreated patients with mCRC who previously achieved sustained disease control.

## 1 Introduction

Chemotherapy combined with targeted therapy has improved survival outcomes for patients with metastatic colorectal cancer (mCRC). However, treatment options remain limited after failure of standard therapies [1,2,3]. Reintroducing a regimen discontinued solely because of cumulative toxicity may provide predictable clinical benefit once treatment-related adverse effects have resolved [4]. In contrast, rechallenge after acquired resistance may involve a different biological mechanism. Tumor progression is driven by the evolution of heterogeneous clonal populations, and intervening therapies may suppress resistant clones, allowing previously sensitive tumor cell populations to re-emerge and restore drug susceptibility over time [5,6].

Oxaliplatin-based combination chemotherapy is a well-established and effective first-line treatment for mCRC [7,8]. However, evidence regarding oxaliplatin rechallenge remains limited. Previous studies have reported objective response rates (ORRs) of approximately 20% and disease control rates (DCRs) of around 40% with oxaliplatin-based retreatment regimens. Reported median progression-free survival (PFS) ranges from 1.6 to 6.0 months, while median overall survival (OS) varies between 2 and 11 months [9,10]. These heterogeneous outcomes underscore the variability and uncertain clinical benefit of oxaliplatin rechallenge in heavily pretreated patients. In addition, most available evidence is derived from retrospective studies, and the impact of rechallenge strategies on survival outcomes remains inadequately defined [5].

The potential benefits of cetuximab rechallenge in patients with refractory mCRC have been recently highlighted, particularly those with RAS and BRAF wild-type tumors identified via liquid rebiopsy after secondary resistance to first-line cetuximab [11,12]. A phase II trial showed that cetuximab plus irinotecan rechallenge achieved a DCR of 54%, with longer PFS in patients without RAS mutations in circulating tumor DNA (ctDNA) [13]. Similarly, a Japanese phase II study reported a median PFS of 2.4 months and OS of 8.2 months with the same regimen [14]. This variability highlights the uncertain clinical benefit of this approach in heavily pretreated patients. Importantly, most available evidence regarding these retreatment strategies is derived from retrospective studies, and prospective clinical data remain limited. Furthermore, the comparative efficacy of chemotherapy reintroduction alone versus in combination with targeted therapy in the late-line setting has not been systematically evaluated.

To address these knowledge gaps, this prospective, single-arm, phase II study aimed to evaluate the efficacy and safety of rechallenge strategies using previously effective chemotherapy regimens, either alone or combined with targeted agents (bevacizumab or cetuximab), in heavily pretreated patients with refractory mCRC.

## 2 Methods

### 2.1 Study Design and Patients

This investigator-initiated, single-center, single-arm phase II trial (ClinicalTrials.gov Identifier: NCT03485027) was conducted at Fudan University Shanghai Cancer Center (FUSCC) following approval from the Institutional Review Board (Approval Number: 1709176-9-1710). The study adhered to the ethical principles outlined in the Declaration of Helsinki and complied with all relevant local laws and regulations. Between February 2018 and December 2022, eligible patients with histologically confirmed mCRC who had failed at least two prior lines of systemic chemotherapy were enrolled. Eligible participants received rechallenge regimens consisting of previously administered chemotherapy, with or without the addition of targeted therapies. Prior to enrollment, all participants provided written informed consent.

Patients were eligible for inclusion if they were aged 18–80 years and had histologically confirmed mCRC with treatment failure after at least two lines of standard systemic chemotherapy, which included oxaliplatin, fluorouracil, and irinotecan. Treatment regimens combined with cetuximab or bevacizumab were permitted. Treatment failure was defined as disease progression during therapy or within three months after the last dose, or intolerance to toxicities. Additionally, eligible patients must have demonstrated a PFS of at least four months during the prior therapy which was selected for rechallenge, without any unresolved toxicity. Other criteria included at least one measurable lesion, as defined by Response Evaluation Criteria in Solid Tumors (RECIST) version 1.1, and adequate hematologic, hepatic, and renal function within seven days of screening. An Eastern Cooperative Oncology Group (ECOG) performance status of 0–2 was required.

Key exclusion criteria included receipt of any other systemic anticancer therapy within three weeks before study enrollment and unresolved grade 3 or 4 chemotherapy-related toxicities. Patients were also excluded if they had additional malignancies within the previous five years, except for adequately treated *in situ* cervical carcinoma or basal cell carcinoma. Other exclusion criteria included symptomatic intracranial or meningeal metastases, uncontrolled pleural or peritoneal effusions, and any severe or life-threatening comorbid condition that, in the investigator’s judgment, could compromise patient safety or interfere with protocol adherence.

### 2.2 Treatment

The rechallenge regimens consisted of oxaliplatin- or irinotecan-based chemotherapy, selected based on the longest interval since the prior administration of these agents. Combination regimens included XELOX (capecitabine plus oxaliplatin; comprising intravenous oxaliplatin 130 mg/m^2^ on day 1 and oral capecitabine 1000 mg/m^2^ twice daily for 14 consecutive days), with or without bevacizumab 7.5 mg/kg intravenously on day 1, repeated every three weeks; FOLFOX (oxaliplatin, leucovorin, and 5-fluorouracil [5-FU]; comprising intravenous oxaliplatin 85 mg/m^2^, calcium folinate 400 mg/m^2^, and a 5-FU bolus of 400 mg/m^2^ on day 1, followed by continuous intravenous infusion of 5-FU 2.4 g/m^2^ over 46 h), with or without bevacizumab 5 mg/kg or cetuximab 500 mg/m^2^, repeated every two weeks; and FOLFIRI (irinotecan, leucovorin, and 5-FU; comprising intravenous irinotecan 180 mg/m^2^, calcium folinate 400 mg/m^2^, and a 5-FU bolus of 400 mg/m^2^ on day 1, followed by continuous infusion of 5-FU 2.4 g/m^2^ over 46 h), with or without bevacizumab 5 mg/kg or cetuximab 500 mg/m^2^, repeated every two weeks. Irinotecan-containing regimen was administered 180 mg/m^2^ on day 1, with or without bevacizumab 5 mg/kg or cetuximab 500 mg/m^2^ every two weeks. Raltitrexed-containing regimens included raltitrexed 3 mg/m^2^ intravenously on day 1, with or without bevacizumab 7.5 mg/kg, every three weeks, or raltitrexed 2 mg/m^2^ with or without cetuximab 500 mg/m^2^ every two weeks. Treatment was continued until radiologically confirmed disease progression, the occurrence of unacceptable adverse events (AEs), or withdrawal of consent. All targeted agents used in this study were originator drugs, including bevacizumab (Avastin; Roche, Basel, Switzerland) and cetuximab (Erbitux; Merck KGaA, Darmstadt, Germany).

### 2.3 Assessments and Endpoints

Laboratory evaluations were performed before each treatment cycle. Tumor assessments were conducted at baseline and every six weeks thereafter using contrast-enhanced computed tomography (CT) or magnetic resonance imaging (MRI), and all efficacy evaluations were investigator-assessed by the same radiologist according to RECIST version 1.1 [15]. AEs were graded according to the Common Terminology Criteria for Adverse Events (CTCAE), version 4.0, and all AEs occurring within 28 days of the last dose were recorded. In cases of grade 3 or 4 AEs, treatment was suspended until toxicity resolved or improved to an acceptable level.

Serious adverse events (SAEs) were defined as any untoward medical occurrence that at any dose resulted in death, was life-threatening, required inpatient hospitalization or prolongation of existing hospitalization, resulted in persistent or significant disability/incapacity, or congenital anomaly/birth defect, or was considered an important medical event. Important medical events referred to events that might not immediately result in death or hospitalization but could jeopardize the patient or require intervention to prevent serious outcomes. Any suspected transmission of an infectious agent via the investigational drug was also classified as an SAE.

The primary endpoint was PFS, defined as the time from enrollment to either disease progression or death from any cause, with censoring at the date of the last follow-up for patients without progression. Secondary endpoints included ORR, defined as the proportion of patients achieving complete response (CR) or partial response (PR); DCR, defined as the proportion achieving CR, PR, or stable disease (SD); OS, defined as the time from enrollment to death from any cause; and the incidence and severity of AEs in all enrolled patients.

### 2.4 Statistical Analysis

The sample size was determined based on the primary endpoint of PFS. The null hypothesis assumed a historical median PFS of 1.5 months following the failure of front-line therapy, based on the placebo arms of the landmark phase III CORRECT and RECOURSE trials [16,17]. The alternative hypothesis assumed that the rechallenge strategy would prolong the median PFS to 3.5 months [18]. Therefore, the pre-defined efficacy threshold for success was a median PFS of at least 3.5 months. The planned enrollment period for this study was two years, followed by a one-year follow-up. With 80% power and a two-sided alpha level of 0.05, 33 patients were required; allowing for an estimated 20% of drop-out, the planned sample size was 42 patients.

Survival analyses were performed on the full analysis set (FAS), which included patients who received at least one cycle of the assigned chemotherapy regimen and had at least one post-baseline tumor response evaluation. Safety analyses included all patients who received at least one dose of study treatment and underwent at least one safety assessment. Hazard ratios (HRs) and 95% confidence intervals (CIs) were calculated using the Cox proportional hazards model. Survival curves for PFS and OS were generated using the Kaplan-Meier method, with censoring applied to patients who were alive at the time of the last follow-up. Statistical tests were two-sided, with *p* values < 0.05 considered significant. Subgroup analyses were exploratory and were not pre-specified due to the small sample size and the study’s exploratory nature. Statistical analyses were performed using SPSS software, version 22.0 (SPSS Inc., Chicago, IL, USA).

## 3 Results

### 3.1 Baseline Characteristics of Patients

**Figure 1:**
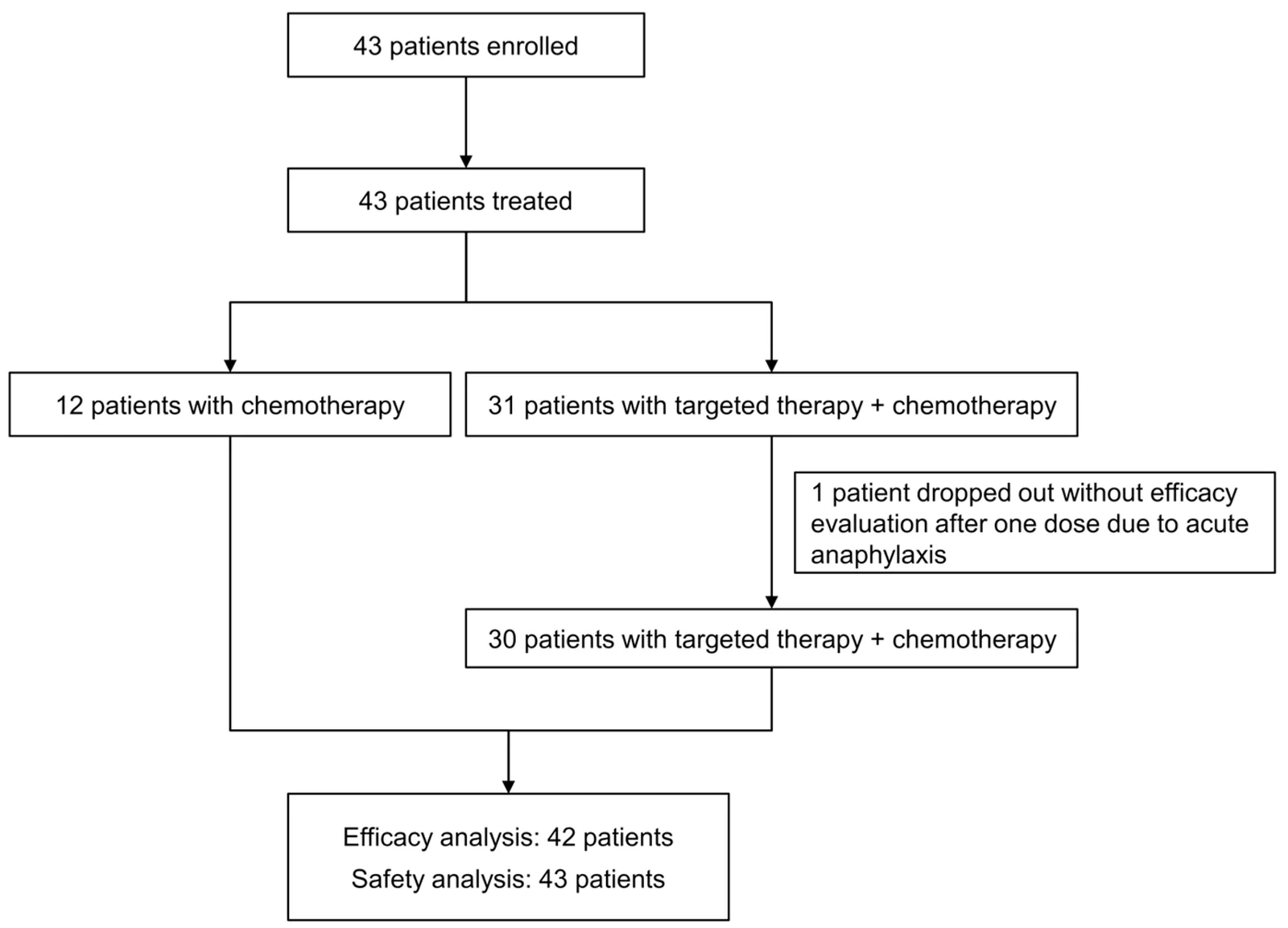
Trial flowchart.

Between 3 February 2018, and 10 December 2022, 43 eligible patients with mCRC were enrolled in the study. The median age was 59 years (range 37–82 years). Baseline demographic and clinical characteristics are summarized in Table 1. All patients received at least one cycle of the rechallenge regimen, and 42 patients underwent response evaluation (Fig. 1). By the cut-off date of 1 July 2023, the median follow-up duration was 26.3 months (range 4.9–64.9 months), and all enrolled patients had discontinued the study treatment. One patient discontinued the rechallenge regimen after experiencing oxaliplatin-related acute anaphylaxis, while the remaining 42 patients discontinued treatment due to disease progression.

**Table 1:** Baseline characteristics of patients.

Characteristic	All Patients (n = 43)
**Age (years), n (%)**	
** <65**	34 (79.1%)
** ≥65**	9 (20.9%)
**Sex, n (%)**	
** Male**	22 (51.2%)
** Female**	21 (48.8%)
**ECOG performance status, n (%)**	
** 0–1**	30 (69.8%)
** 2**	13 (30.2%)
**Primary tumor site, n (%)**	
** Left colon**	26 (60.5%)
** Right colon**	17 (39.5%)
**KRAS/NRAS mutation status, n (%)**	
** Mutated**	25 (58.1%)
** Wild-type**	18 (41.9%)
**Number of metastatic sites, n (%)**	
** 1**	13 (30.2%)
** ≥2**	30 (69.8%)
**Metastatic site, n (%)**	
** Liver**	20 (46.5%)
** Lung**	12 (27.9%)
** Lymph nodes**	18 (41.9%)
** Peritoneum**	15 (34.9%)
** Bone**	8 (18.6%)
**Time interval between rechallenge and prior administration, n (%)**	
** ≥1 year**	29 (67.4%)
** <1 year**	14 (32.6%)
**Treatment line of rechallenge regimen in the prior setting, n (%)**	
** First-line**	17 (39.5%)
** Second-line**	26 (60.5%)
**Response to rechallenge regimen in the prior setting, n (%)**	
** Partial response**	9 (20.9%)
** Stable disease**	34 (79.1%)
**Type of rechallenge regimen, n (%)**	
** Chemotherapy alone**	12 (27.9%)
** Chemotherapy plus bevacizumab**	20 (46.5%)
** Chemotherapy plus cetuximab**	11 (25.6%)
**Treatment line of the rechallenge regimen, n (%)**	
** Third-line**	5 (11.6%)
** Fourth-line or later**	38 (88.4%)

ECOG, Eastern Cooperative Oncology Group; KRAS, kirsten rat sarcoma viral oncogene homolog; NRAS, neuroblastoma rat sarcoma viral oncogene homolog. One patient was reported as aged 82 years during follow-up based on self-reported information; however, the registered age documented on the official identification provided at screening met the study inclusion criteria. Time interval refers to the interval between the last administration of the rechallenged regimen and study enrollment, rather than a general chemotherapy-free interval.

### 3.2 The Rechallenge Regimens

All patients received rechallenge treatment, with 12 (27.9%) patients receiving chemotherapy alone and 31 (72.1%) patients receiving chemotherapy combined with targeted agents. Among those who received chemotherapy alone, seven patients were treated with the XELOX regimen, and five patients received irinotecan-based regimens, including four who received irinotecan monotherapy and one who received the FOLFIRI regimen. In the combination therapy group, 20 patients received bevacizumab-based regimens, comprising seven patients treated with bevacizumab plus XELOX, 10 patients treated with bevacizumab plus irinotecan, and three patients treated with bevacizumab combined with raltitrexed. An additional 11 patients received cetuximab in combination with irinotecan. All 11 patients who received cetuximab-based rechallenge were confirmed to have KRAS/NRAS and BRAF wild-type tumors based on available tissue molecular testing. The median duration of rechallenge treatment for all patients was 3.33 months (range 0.70–19.57 months).

### 3.3 Primary Endpoint

**Figure 2:**
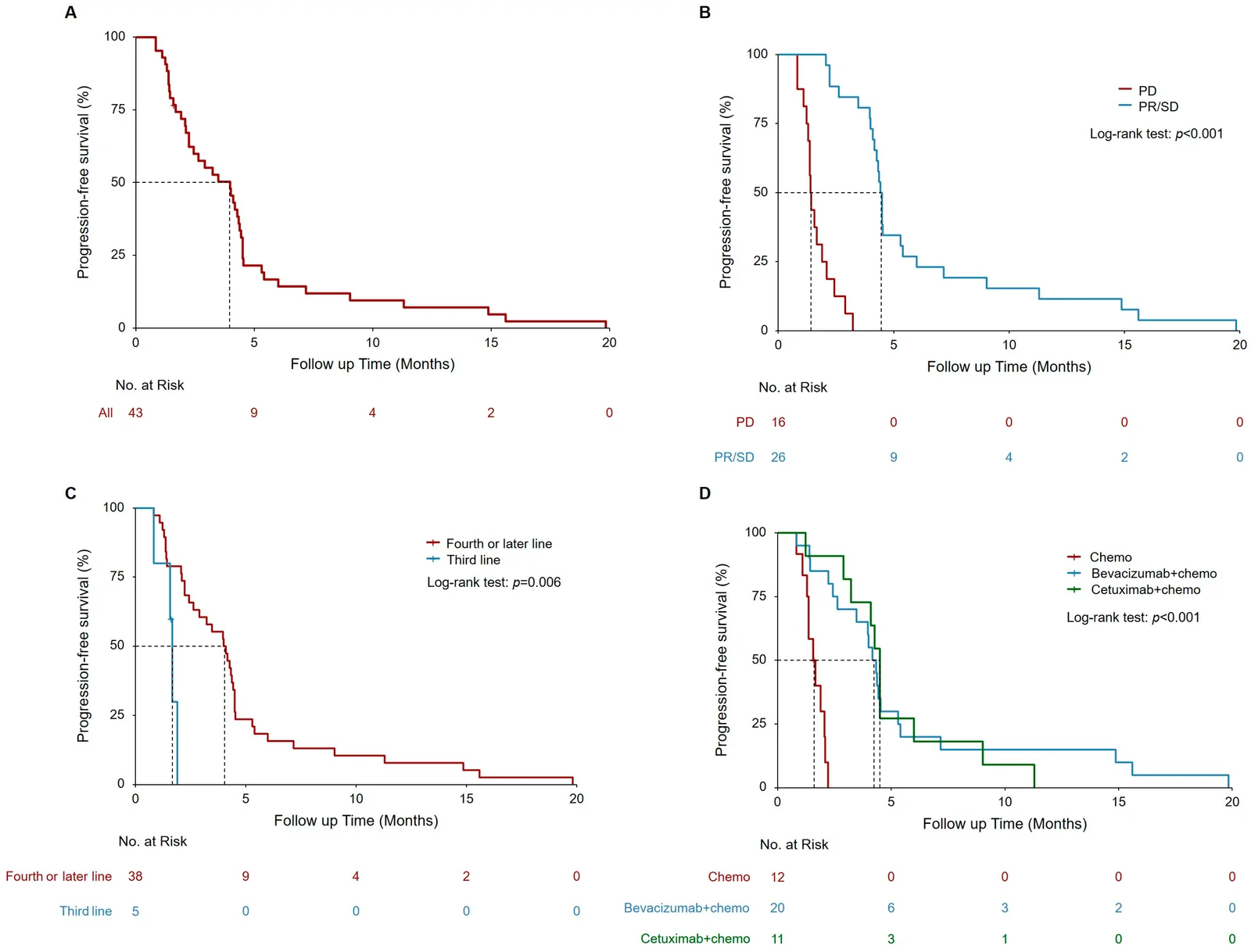
**Kaplan-Meier curves of investigator-assessed progression-free survival (PFS).** (**A**) PFS in the overall study population. (**B**) PFS according to the best tumor response, comparing patients who achieved partial response (PR) or stable disease (SD) versus those with progressive disease (PD). (**C**) PFS according to treatment line, comparing patients receiving retreatment as third-line therapy versus later-line therapy. (**D**) PFS according to retreatment strategy, comparing chemotherapy alone versus chemotherapy combined with cetuximab or bevacizumab.

In the FAS, PFS events occurred in 42 patients. The median PFS, as assessed by investigators, was 3.97 months (95% CI: 2.46–5.48 months) (Fig. 2A), exceeding the predefined efficacy threshold of 3.5 months and therefore meeting the pre-specified primary endpoint assumptions of the study. The estimated PFS rate at three months was 55.2% (95% CI: 40.1–70.3%), while the estimated six-month PFS rate was 16.8% (95% CI: 5.4–28.2%). Subgroup analyses revealed that patients who achieved PR or SD had longer median PFS compared with those who experienced progressive disease (PD) (4.43 vs. 1.40 months; HR = 0.049, 95% CI: 0.016–0.155) (Fig. 2B). Patients receiving rechallenge regimens as third-line therapy had shorter PFS compared with those receiving these regimens as fourth-line or later treatments (1.67 vs. 4.00 months) (Fig. 2C). Furthermore, patients who received combination chemotherapy with targeted therapy had longer PFS compared with those who received chemotherapy alone (median PFS: 4.17 months for chemotherapy plus bevacizumab, and 4.50 months for chemotherapy plus cetuximab, vs. 1.57 months for chemotherapy alone) (Fig. 2D).

### 3.4 Secondary Endpoints

Of the 43 enrolled patients, 42 underwent response evaluation, as one patient discontinued treatment after a single dose of rechallenged oxaliplatin-based chemotherapy due to an acute allergic reaction. Among the evaluable population, one patient (2.4%) achieved a PR, resulting in an ORR of 2.4%. SD was observed in 25 patients, while 16 patients experienced PD, yielding a DCR of 61.9% (26/42). Patients with a treatment interval of more than one year between the prior regimen and rechallenge demonstrated a higher DCR compared with those with an interval of less than one year (72.4% vs. 38.5%). Regarding rechallenge regimens, combination chemotherapy with targeted therapy was associated with higher DCRs compared to chemotherapy alone (80.0% for bevacizumab-based regimens and 72.7% for cetuximab-based regimens vs. 18.2% for chemotherapy alone). Irinotecan-based regimens showed higher DCR compared with oxaliplatin-based and raltitrexed-based regimens (72.0% vs. 57.1% and 0.0%, respectively). Detailed subgroup analyses of DCRs are presented in Table 2.

**Table 2:** Subgroup analyses of disease control rates (DCRs) (n = 42).

Subgroup	DCR (%)	*p* Value
**Time interval between rechallenge and prior administration**		
** <1 year**	38.5% (5/13)	0.036
** ≥1 year**	72.4% (21/29)	
**Response to rechallenge regimen in the prior setting**		0.740
** Partial response**	66.6% (6/9)	
** Stable disease**	60.6% (20/33)	
**Treatment line of rechallenge regimen in the prior setting**		0.735
** First-line**	58.8% (10/17)	
** Second-line**	64.0% (16/25)	
**Type of rechallenge regimen**		0.002
** Chemotherapy alone**	18.2% (2/11)	
** Chemotherapy plus bevacizumab**	80.0% (16/20)	
** Chemotherapy plus cetuximab**	72.7% (8/11)	
**Chemotherapeutic agents in rechallenge regimens**		0.048
** Oxaliplatin-based**	57.1% (8/14)	
** Irinotecan-based**	72.0% (18/25)	
** Raltitrexed-based**	0.0 (0/3)	
**Treatment line of rechallenge regimen**		0.007
** Third-line**	0 (0/4)	
** Fourth-line or later**	68.4% (26/38)	
**Primary tumor site**		0.735
** Right colon**	58.8% (10/17)	
** Left colon**	64.0% (16/25)	

**Figure 3:**
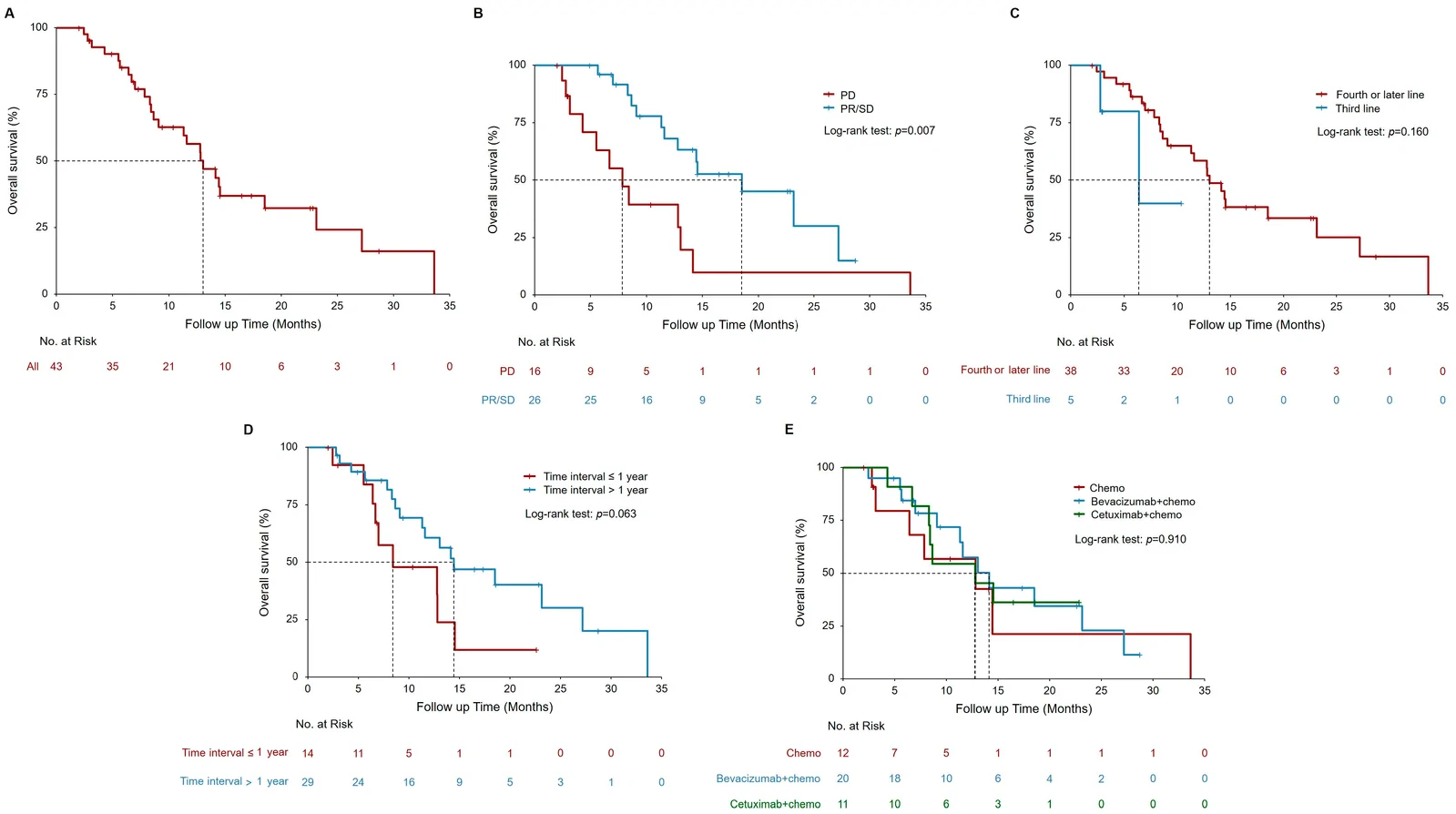
Kaplan-Meier curves of investigator-assessed overall survival (OS). (**A**) OS in the overall study population. (**B**) OS according to best tumor response, comparing patients who achieved partial response (PR) or stable disease (SD) versus those with progressive disease (PD). (**C**) OS according to treatment line, comparing patients receiving retreatment as third-line therapy versus later-line therapy. (**D**) OS according to the interval between prior treatment and retreatment (≤1 year vs. >1 year). (**E**) OS according to retreatment strategy, comparing chemotherapy alone versus chemotherapy combined with cetuximab or bevacizumab.

At the follow-up cutoff date, 26 patients had died. The median OS in the FAS was 13.03 months (95% CI: 9.68–16.38) (Fig. 3A). The estimated OS rates at 6 and 12 months were 85.1% (95% CI: 74.1–96.1%) and 56.5% (95% CI: 40.2–72.8%), respectively. Subgroup analyses showed results consistent with those observed for PFS. Patients who achieved PR/SD had longer OS compared to those with PD (median OS: 18.50 months vs. 7.83 months) (Fig. 3B). Patients receiving rechallenge as third-line therapy had numerically shorter OS compared to those treated at fourth-line or beyond (6.40 vs. 13.03 months) (Fig. 3C). Similarly, patients with a rechallenge interval of more than one year showed a trend toward improved OS compared with those with a shorter interval (14.43 vs. 8.40 months) (Fig. 3D). There was no significant difference in OS between the types of rechallenge regimens (chemotherapy alone vs. chemotherapy combined with targeted therapy) (Fig. 3E).

### 3.5 Safety

The most common AEs of any grade were white blood cell count decreased (90.7%), neutrophil count decreased (83.7%), and anemia (79.1%). Grade 3–4 AEs occurred in 39.5% (17/43) of patients, with the most frequent being neutrophil count decreased (30.2%), white blood cell count decreased (25.6%), and platelet count decreased (20.9%) (Table 3). Dose reductions due to grade 3–4 AEs were required in eight patients (18.6%). Treatment interruptions due to AEs occurred in six patients (14.0%) and included platelet count decreased, oxaliplatin-related allergic reaction, hand-foot syndrome, and diarrhea. One patient discontinued treatment after experiencing a grade 3 oxaliplatin-related allergic reaction following the first cycle of rechallenge. No treatment-related deaths occurred during the study. All reported deaths were due to tumor progression. Additionally, no SAEs were observed during the treatment period.

**Table 3:** Adverse events (N = 43).

Events, n (%)	Grade 1–2	Grade 3	Grade 4
**Hematological**			
** Anemia**	32 (74.4%)	1 (2.3%)	1 (2.3%)
** White blood cell count decreased**	28 (65.1%)	10 (23.3%)	1 (2.3%)
** Neutrophil count decreased**	23 (53.5%)	11 (25.6%)	2 (4.7%)
** Platelet count decreased**	16 (37.2%)	6 (14.0%)	3 (7.0%)
**Non-hematological**			
** Nausea**	25 (58.1%)	1 (2.3%)	0
** Anorexia**	20 (46.5%)	0	0
** Aspartate aminotransferase increased**	19 (44.2%)	1 (2.3%)	0
** Alanine aminotransferase increased**	17 (39.5%)	1 (2.3%)	0
** Fatigue**	16 (37.2%)	1 (2.3%)	0
** Oral mucositis**	15 (34.9%)	0	0
** Peripheral neuropathy**	14 (32.6%)	0	0
** Hand-foot syndrome**	11 (25.6%)	1 (2.3%)	0
** Diarrhea**	9 (20.9%)	2 (4.7%)	0
** Blood bilirubin increased**	9 (20.9%)	1 (2.3%)	0
** Vomiting**	5 (11.6%)	3 (7.0%)	0
** Allergic reaction**	2 (4.7%)	1 (2.3%)	0

Percentages are based on the entire cohort; the format represents *n* (sample size)/*N* (total number of patients, *N* = 43).

## 4 Discussion

This exploratory phase II study evaluated the efficacy and safety of retreatment with previously administered regimens in heavily pretreated patients with mCRC. To our knowledge, this is the first prospective study to evaluate multiple retreatment strategies, including different chemotherapy regimens with or without targeted therapy, within a single trial. In the FAS, the median PFS was 3.97 months, and the DCR reached 61.9%, suggesting potential clinical activity in selected patients. Although the ORR was low (2.4%), durable disease stabilization was observed in a substantial proportion of patients. The median OS was 13.03 months. In addition, the safety profile was generally manageable, with no unexpected toxicities, treatment-related deaths, or SAEs observed.

For mCRC, standard first- and second-line treatments generally consist of oxaliplatin- or irinotecan-based chemotherapy regimens, including FOLFOX, XELOX, and FOLFIRI, frequently combined with targeted agents such as bevacizumab, cetuximab, or panitumumab to improve clinical outcomes [19,20]. After failure of standard therapies, available late-line treatment options remain limited and mainly include regorafenib, fruquintinib, and TAS-102, administered alone or in combination with bevacizumab [21]. Nevertheless, many patients retain adequate ECOG performance status after progression on third-line therapy and may still be candidates for additional systemic treatment [22,23,24], which has led to increasing interest in retreatment strategies in carefully selected patients [25,26,27,28]. In the present study, the DCR of 61.9% observed with retreatment regimens was numerically comparable to the DCR reported with regorafenib in the phase III CORRECT and CONCUR trials [16,29], and appeared higher than that reported with TAS-102 monotherapy in the RECOURSE and TERRA studies [17,30]. However, these cross-trial comparisons are not statistically valid because of substantial differences in study populations, inclusion criteria, and trial designs. In particular, patients enrolled in the present study represented a highly selected population with prior sensitivity to the rechallenged regimen.

Previous studies on retreatment strategies in mCRC have mainly focused on chemotherapy regimens that were discontinued before treatment failure, often as part of a “stop-and-go” strategy. In a multicenter phase II randomized trial, Matsuda et al. [9] compared biweekly XELOX with the standard triweekly XELOX regimen as retreatment therapy and reported median PFS and OS of 3.3 and 9.2 months in the biweekly group and 4.3 and 12.1 months in the triweekly group, respectively, without significant differences in efficacy or safety. Similarly, the OPTIMOX1 study reported a DCR of 49.4% with FOLFOX reintroduction [31]. The RE-OPEN trial evaluating oxaliplatin retreatment reported an ORR of 6.1%, while 33.3% of patients achieved stable disease at 12 weeks [32]. In the present study, multiple chemotherapy regimens were evaluated with or without targeted therapy. Oxaliplatin-based retreatment achieved a DCR of 57.1%, which was generally consistent with previous reports. Irinotecan-based regimens demonstrated a numerically higher DCR of 72.0%. In addition, patients treated with chemotherapy combined with bevacizumab or cetuximab showed improved DCR and longer PFS compared with chemotherapy alone. However, because these subgroup analyses were exploratory and non-randomized, these findings should be interpreted cautiously.

RAS wild-type mCRC because previously suppressed EGFR-sensitive tumor clones may re-emerge over time, potentially restoring sensitivity to EGFR blockade [33]. Cremolini et al. [13] evaluated cetuximab plus irinotecan retreatment in patients with RAS/BRAF wild-type mCRC and reported an ORR of 21%, a DCR of 54%, and median PFS and OS of 4.0 and 12.5 months, respectively. Similarly, the phase II CAVE trial demonstrated that cetuximab combined with avelumab was an active and manageable retreatment strategy in this patient population [34]. In the present study, 11 patients received cetuximab-based retreatment combined with irinotecan-containing chemotherapy, achieving an ORR of 9.1% and a DCR of 72.7%. These findings were generally consistent with previous reports, including the JACCRO CC-08 trial, which reported a DCR of 55.9%, median PFS of 2.4 months, and median OS of 8.2 months [14]. Notably, the JACCRO CC-08 study suggested that longer cetuximab-free intervals were associated with improved outcomes, indicating that treatment-free interval may be an important clinical selection factor for anti-EGFR retreatment [14]. Emerging evidence also highlights the importance of molecular selection before anti-EGFR re-exposure. In a pooled analysis of four prospective trials, Ciardiello et al. [35] reported that patients with ctDNA-confirmed RAS/BRAF wild-type tumors achieved a median PFS of 4.0 months and median OS of 13.1 months following anti-EGFR retreatment. These findings suggest that ctDNA analysis may help identify patients most likely to benefit from anti-EGFR retreatment by dynamically monitoring resistant tumor clones. However, because ctDNA testing was not routinely performed in the present study, these findings should be interpreted cautiously.

While cetuximab rechallenge has been widely studied, few reports have evaluated bevacizumab combined with chemotherapy. In our study, bevacizumab-based combinations achieved a DCR of 80.0% (16/20) and a median PFS of 4.17 months, demonstrating that non-cetuximab rechallenge strategies can also be effective. In the current study, this combination continued to show potential efficacy as a later-line rechallenge option. Subgroup analysis showed that longer intervals (≥1 year) between prior treatment and rechallenge were associated with higher DCR, suggesting prolonged chemotherapy-free interval may lead to restored tumor sensitivity. These results emphasize the importance of considering treatment-free intervals when selecting rechallenge strategies for refractory mCRC.

In our study, the AEs associated with rechallenge regimens were generally well tolerated. Grade 3/4 non-hematological AEs were rare, with all occurring at rates below 5%, and no grade 4 non-hematological events were observed. One notable AE leading to discontinuation of oxaliplatin was anaphylaxis, a known concern during oxaliplatin rechallenge, which is reported to occur in approximately 0.5% of patients in previous studies, with the risk increasing after repeated cycles [36]. Based on clinical experience at our center, administering a full dose (15–20 mg) of dexamethasone via slow intravenous drip rather than direct injection can significantly reduce the risk of hypersensitivity reactions. In this study, three patients experienced allergic reactions: two cases were mild, presenting with skin rash and itching, while one patient developed dyspnea and chest tightness, with rapid improvement with treatment, and no life-threatening events occurred. Importantly, no SAEs or treatment-related deaths were reported during the study period, supporting the overall safety and feasibility of rechallenge strategies in heavily pretreated patients.

Our study has several limitations that should be acknowledged. This phase II study was non-randomized and had a relatively small sample size, with all enrolled patients recruited from a single center, which may limit the generalizability of the findings. The lack of a randomized control group limited the ability to directly compare the efficacy of different rechallenge strategies or determine the independent contribution of targeted therapy. Therefore, these results should be considered preliminary, with further large-scale studies with longer follow-up required to validate results. Additionally, none of the patients included in this study had achieved a CR to their prior treatment regimens, preventing us from drawing any conclusions about the efficacy of rechallenge therapy in this specific subgroup. However, it is important to note that the CR population is exceedingly rare in studies on rechallenge strategies, as highlighted in previous reports [14]. In selected patients with refractory mCRC who previously achieved disease control with oxaliplatin- or irinotecan-based regimens and had an adequate treatment-free interval, chemotherapy rechallenge with or without targeted therapy demonstrated modest disease control and manageable toxicity. However, these findings should be considered exploratory and require further validation in larger randomized studies with molecularly defined patient selection. Because treatment allocation was not randomized, differences in baseline characteristics, molecular status, prior treatment sensitivity, and treatment-free interval may have influenced outcomes between treatment groups. Therefore, the improved efficacy observed with targeted therapy-containing regimens should be interpreted as exploratory. Besides, information regarding subsequent therapies after study treatment was incomplete because many patients were lost to follow-up after disease progression and study withdrawal, which may have influenced OS outcomes. Furthermore, comprehensive biomarker assessments and patient-reported quality-of-life data were not routinely collected, which may limit the clinical interpretation and generalizability of the findings.

## 5 Couclusion

This phase II study suggests that rechallenge regimens, including chemotherapy alone or in combination with targeted therapy previously administered in earlier-line treatment, may provide potential clinical benefit in selected heavily pretreated patients with mCRC who previously achieved disease control and maintained a sufficient treatment-free interval. Rechallenge therapy was feasible and manageable in selected patients, although clinically relevant hematologic toxicities were common. This study provides prospective evidence supporting the feasibility of rechallenge strategies in carefully selected patients in the late-line setting. Larger randomized studies are warranted to further validate these findings and clarify the optimal patient selection criteria and role of rechallenge therapy in clinical practice.

## Data Availability

The authors confirm that the data supporting the findings of this study are available within the article.

## Abbreviations

AEs	adverse events
ECOG	eastern cooperative oncology group
CT	computed tomography
CR	complete response
CIs	confidence intervals
CTCAE	common terminology criteria for adverse events
DCR	disease control rate
HRs	hazard ratios
MRI	magnetic resonance imaging
mCRC	metastatic colorectal cancer
ORR	objective response rate
OS	overall survival
PFS	progression-free survival
PR	partial response
PD	progressive disease
SD	stable disease

## References

[1] Abedizadeh R , Majidi F , Khorasani HR , Abedi H , Sabour D . Colorectal cancer: A comprehensive review of carcinogenesis, diagnosis, and novel strategies for classified treatments. Cancer Metastasis Rev. 2024; 43( 2): 729– 53. doi:10.1007/s10555-023-10158-3. 38112903

[2] Önder AH , Çatli MM . Colorectal cancer treatment commentary review. Eur J Cancer. 2025; 230: 115809. doi:10.1016/j.ejca.2025.115809. 41014853

[3] Underwood PW , Ruff SM , Pawlik TM . Update on targeted therapy and immunotherapy for metastatic colorectal cancer. Cells. 2024; 13( 3): 245. doi:10.3390/cells13030245. 38334637 PMC10854977

[4] Amatu A , Mauri G , Tosi F , Bencardino K , Bonazzina E , Gori V , et al. Efficacy of retreatment with oxaliplatin-based regimens in metastatic colorectal cancer patients: The RETROX-CRC retrospective study. Cancers. 2022; 14( 5): 1197. doi:10.3390/cancers14051197. 35267504 PMC8909235

[5] Mauri G , Gori V , Bonazzina E , Amatu A , Tosi F , Bencardino K , et al. Oxaliplatin retreatment in metastatic colorectal cancer: Systematic review and future research opportunities. Cancer Treat Rev. 2020; 91: 102112. doi:10.1016/j.ctrv.2020.102112. 33091698

[6] Hack J , Crabb SJ . Platinum-based chemotherapy ‘rechallenge’ in advanced non-ovarian solid malignancies. Clin Oncol. 2022; 34( 8): e329– 44. doi:10.1016/j.clon.2022.02.015. 35282934

[7] Kawai S , Takeshima N , Hayasaka Y , Notsu A , Yamazaki M , Kawabata T , et al. Comparison of irinotecan and oxaliplatin as the first-line therapies for metastatic colorectal cancer: A meta-analysis. BMC Cancer. 2021; 21( 1): 116. doi:10.1186/s12885-021-07823-7. 33541293 PMC7863255

[8] Fan S , Zhao Z , Wang H , Wang H , Niu W . Efficacy and safety of oxaliplatin-based chemotherapy as first-line treatment in elderly patients with metastatic colorectal cancer: A meta-analysis. Front Oncol. 2025; 15: 1567732. doi:10.3389/fonc.2025.1567732. 40260292 PMC12009691

[9] Matsuda C , Honda M , Tanaka C , Fukunaga M , Ishibashi K , Munemoto Y , et al. Multicenter randomized phase II clinical trial of oxaliplatin reintroduction as a third- or later-line therapy for metastatic colorectal cancer-biweekly versus standard triweekly XELOX (The ORION Study). Int J Clin Oncol. 2016; 21( 3): 566– 72. doi:10.1007/s10147-015-0911-7. 26475356

[10] Costa T , Nuñez J , Felismino T , Boente L , Mello C . REOX: Evaluation of the efficacy of retreatment with an oxaliplatin-containing regimen in metastatic colorectal cancer: A retrospective single-center study. Clin Color Cancer. 2017; 16( 4): 316– 23. doi:10.1016/j.clcc.2017.03.002. 28392022

[11] Mariani S , Puzzoni M , Giampieri R , Ziranu P , Pusceddu V , Donisi C , et al. Liquid biopsy-driven cetuximab rechallenge strategy in molecularly selected metastatic colorectal cancer patients. Front Oncol. 2022; 12: 852583. doi:10.3389/fonc.2022.852583. 35530345 PMC9068964

[12] Nishida T , Doi T . Rechallenge of drugs in the era of targeted therapy. Lancet Oncol. 2013; 14( 12): 1143– 5. doi:10.1016/S1470-2045(13)70494-7. 24140185

[13] Cremolini C , Rossini D , Dell’Aquila E , Lonardi S , Conca E , del Re M , et al. Rechallenge for patients with RAS and BRAF wild-type metastatic colorectal cancer with acquired resistance to first-line cetuximab and irinotecan: A phase 2 single-arm clinical trial. JAMA Oncol. 2019; 5( 3): 343– 50. doi:10.1001/jamaoncol.2018.5080. 30476968 PMC6439839

[14] Masuishi T , Tsuji A , Kotaka M , Nakamura M , Kochi M , Takagane A , et al. Phase 2 study of irinotecan plus cetuximab rechallenge as third-line treatment in KRAS wild-type metastatic colorectal cancer: JACCRO CC-08. Br J Cancer. 2020; 123( 10): 1490– 5. doi:10.1038/s41416-020-01042-w. 32863385 PMC7652864

[15] Eisenhauer E , Therasse P , Bogaerts J , Schwartz L , Sargent D , Ford R , et al. New response evaluation criteria in solid tumours: Revised RECIST guideline (version 1.1). Eur J Cancer. 2009; 45( 2): 228– 47. doi:10.1016/j.ejca.2008.10.026. 19097774

[16] Grothey A , Van Cutsem E , Sobrero A , Siena S , Falcone A , Ychou M , et al. Regorafenib monotherapy for previously treated metastatic colorectal cancer (CORRECT): An international, multicentre, randomised, placebo-controlled, phase 3 trial. Lancet. 2013; 381( 9863): 303– 12. doi:10.1016/S0140-6736(12)61900-X. 23177514

[17] Mayer RJ , Van Cutsem E , Falcone A , Yoshino T , Garcia-Carbonero R , Mizunuma N , et al. Randomized trial of TAS-102 for refractory metastatic colorectal cancer. N Engl J Med. 2015; 372( 20): 1909– 19. doi:10.1056/nejmoa1414325. 25970050

[18] Bertocchi P , Aroldi F , Prochilo T , Meriggi F , Beretta GD , Zaniboni A . Chemotherapy rechallenge after regorafenib treatment in metastatic colorectal cancer: Still hope after the last hope? J Chemother. 2017; 29( 2): 102– 5. doi:10.1080/1120009X.2016.1247205. 28032528

[19] Bando H , Ohtsu A , Yoshino T . Therapeutic landscape and future direction of metastatic colorectal cancer. Nat Rev Gastroenterol Hepatol. 2023; 20( 5): 306– 22. doi:10.1038/s41575-022-00736-1. 36670267

[20] Huang M , Yang YN , Li Q , Wang C , Liang L , Zhu X , et al. Induction chemotherapy followed by primary tumor resection did not bring survival benefits in colon cancer patients with asymptomatic primary lesion and synchronous unresectable metastases. Front Oncol. 2022; 12: 747124. doi:10.3389/fonc.2022.747124. 35174078 PMC8841852

[21] Moriwaki T , Fukuoka S , Taniguchi H , Takashima A , Kumekawa Y , Kajiwara T , et al. Propensity score analysis of regorafenib versus trifluridine/tipiracil in patients with metastatic colorectal cancer refractory to standard chemotherapy (REGOTAS): A Japanese society for cancer of the colon and rectum multicenter observational study. Oncologist. 2018; 23( 1): 7– 15. doi:10.1634/theoncologist.2017-0275. 28894015 PMC5759812

[22] Bekaii-Saab T , Kim R , Kim TW , O’Connor JM , Strickler JH , Malka D , et al. Third- or later-line therapy for metastatic colorectal cancer: Reviewing best practice. Clin Colorectal Cancer. 2019; 18( 1): e117– 29. doi:10.1016/j.clcc.2018.11.002. 30598357

[23] Walter T , Hawkins NS , Pollock RF , Colaone F , Shergill S , Ross PJ . Systematic review and network meta-analyses of third-line treatments for metastatic colorectal cancer. J Cancer Res Clin Oncol. 2020; 146( 10): 2575– 87. doi:10.1007/s00432-020-03315-6. 32715436 PMC7467965

[24] Chiang CL , Choi HC , Lam KO , Chan BY , Lee SF , Yeung SY , et al. Real-world treatment patterns and outcomes in refractory metastatic colorectal cancer. Asia Pac J Clncl Oncol. 2019; 15( S2): 5– 13. doi:10.1111/ajco.13114. 30887726

[25] Andrei P , Battuello P , Grasso G , Rovera E , Tesio N , Bardelli A . Integrated approaches for precision oncology in colorectal cancer: The more you know, the better. Semin Cancer Biol. 2022; 84: 199– 213. doi:10.1016/j.semcancer.2021.04.007. 33848627

[26] Huang M , Yang Y , Zhu X , Chen Z , Zhang W , Wang C , et al. A prospective phase II study of raltitrexed combined with S-1 as salvage treatment for patients with refractory metastatic colorectal cancer. Asia Pac J Clin Oncol. 2021; 17( 6): 513– 21. doi:10.1111/ajco.13511. 33567129 PMC9290727

[27] Van Cutsem E , Cervantes A , Adam R , Sobrero A , Van Krieken JH , Aderka D , et al. ESMO consensus guidelines for the management of patients with metastatic colorectal cancer. Ann Oncol. 2016; 27( 8): 1386– 422. doi:10.1093/annonc/mdw235. 27380959

[28] Tonini G , Imperatori M , Vincenzi B , Frezza AM , Santini D . Rechallenge therapy and treatment holiday: Different strategies in management of metastatic colorectal cancer. J Exp Clin Cancer Res. 2013; 32( 1): 92. doi:10.1186/1756-9966-32-92. 24245912 PMC3874688

[29] Li J , Qin S , Xu R , Yau TC , Ma B , Pan H , et al. Regorafenib plus best supportive care versus placebo plus best supportive care in Asian patients with previously treated metastatic colorectal cancer (CONCUR): A randomised, double-blind, placebo-controlled, phase 3 trial. Lancet Oncol. 2015; 16( 6): 619– 29. doi:10.1016/S1470-2045(15)70156-7. 25981818

[30] Xu J , Kim TW , Shen L , Sriuranpong V , Pan H , Xu R , et al. Results of a randomized, double-blind, placebo-controlled, phase III trial of trifluridine/tipiracil (TAS-102) monotherapy in Asian patients with previously treated metastatic colorectal cancer: The TERRA Study. J Clin Oncol. 2018; 36( 4): 350– 8. doi:10.1200/JCO.2017.74.3245. 29215955

[31] Tournigand C , Cervantes A , Figer A , Lledo G , Flesch M , Buyse M , et al. OPTIMOX1: A randomized study of FOLFOX4 or FOLFOX7 with oxaliplatin in a stop-and-Go fashion in advanced colorectal cancer—A GERCOR study. J Clin Oncol. 2006; 24( 3): 394– 400. doi:10.1200/JCO.2005.03.0106. 16421419

[32] Suenaga M , Mizunuma N , Matsusaka S , Shinozaki E , Ozaka M , Ogura M , et al. Phase II study of reintroduction of oxaliplatin for advanced colorectal cancer in patients previously treated with oxaliplatin and irinotecan: RE-OPEN study. Drug Des Devel Ther. 2015; 9: 3099– 108. doi:10.2147/DDDT.S85567. PMC4476424 26124634

[33] Santini D , Vincenzi B , Addeo R , Garufi C , Masi G , Scartozzi M , et al. Cetuximab rechallenge in metastatic colorectal cancer patients: How to come away from acquired resistance? Ann Oncol. 2012; 23( 9): 2313– 8. doi:10.1093/annonc/mdr623. 22396447

[34] Martinelli E , Martini G , Famiglietti V , Troiani T , Napolitano S , Pietrantonio F , et al. Cetuximab rechallenge plus avelumab in pretreated patients with RAS wild-type metastatic colorectal cancer: The phase 2 single-arm clinical CAVE trial. JAMA Oncol. 2021; 7( 10): 1529– 35. doi:10.1001/jamaoncol.2021.2915. 34382998 PMC8531995

[35] Ciardiello D , Martinelli E , Troiani T , Mauri G , Rossini D , Martini G , et al. Anti-EGFR rechallenge in patients with refractory ctDNA RAS/BRAF wt metastatic colorectal cancer: A nonrandomized controlled trial. JAMA Netw Open. 2024; 7( 4): e245635. doi:10.1001/jamanetworkopen.2024.5635. 38592721 PMC11004834

[36] Rassy E , Le Roy F , Smolenschi C , Valéry M , Boige V , Ducreux M , et al. Rechallenge after oxaliplatin-induced hypersensitivity reactions. JAMA Oncol. 2023; 9( 3): 434– 5. doi:10.1001/jamaoncol.2022.7136. 36701137 PMC9880861

